# Quality in the provision of headache care. 1: systematic review of the literature and commentary

**DOI:** 10.1007/s10194-012-0466-1

**Published:** 2012-06-27

**Authors:** Michele Peters, Suraj Perera, Elizabeth Loder, Crispin Jenkinson, Raquel Gil Gouveia, Rigmor Jensen, Zaza Katsarava, Timothy J. Steiner

**Affiliations:** 1 Department of Public Health, University of Oxford, Old Road Campus, Oxford, OX3 7LF UK; 2 Ministry of Health Care and Nutrition, Colombo, Sri Lanka; 3 Division of Headache and Pain, Department of Neurology, Brigham and Women’s Hospital, Boston, MA USA; 4 Headache Outpatient Clinic, Hospital da Luz, Lisbon, Portugal; 5 Department of Neurology, Danish Headache Center, Glostrup Hospital, Glostrup, Denmark; 6 University of Duisburg-Essen, Essen, Germany; 7 Evangelic Hospital, Unna, Germany; 8 Department of Neuroscience, Norwegian University of Science and Technology, Trondheim, Norway; 9 Department of Neuroscience, Imperial College London, London, UK

**Keywords:** Headache, Quality of care, Quality indicators, Systematic review, Global Campaign against Headache

## Abstract

Widely accepted quality indicators for headache care would provide a basis not only for assessment of care but also, and more importantly, for its improvement. The objective of the study was to identify and summarize existing information on such indicators: specifically, did indicators exist, how had they been developed, what aspects of headache care did they relate to and how and with what utility were they being used? A systematic review of the medical literature was performed. A total of 32 articles met criteria for inclusion. We identified 55 existing headache quality indicators of which 37 evaluated processes of headache care. Most were relevant only to specific populations of patients and to care delivered in high-resource settings. Indicators had been used to describe overall quality of headache care at a national level, but not systematically applied to the evaluation and improvement of headache services in other settings. Some studies had evaluated the use of existing disability and quality of life instruments, but their findings had not been incorporated into quality indicators. Existing headache care quality indicators are incomplete and inadequate for purpose. They emphasize processes of care rather than structure or outcomes, and are not widely applicable to different levels and locations of headache care. Furthermore, they do not fully incorporate accepted evidence regarding optimal methods of care. There is a clear need for consensus-based indicators that fully reflect patients’ and public-health priorities. Ideally, these will be valid across cultures and health-care settings.

**Electronic supplementary material:**

The online version of this article (doi:10.1007/s10194-012-0466-1) contains supplementary material, which is available to authorized users.

## Introduction

Despite the high prevalence of recurrent headache disorders—principally migraine and tension-type headache—and the substantial burden of public ill-health they generate [1, 2], there are considerable variations worldwide in the nature, scope, organization, quantity and quality of medical care provided for these illnesses. At the same time, there is good evidence that optimal care is rarely achieved. There are multiple clinical, social and political barriers to both provision of and access to effective headache care, a recent publication by the World Health Organization stated: “The facts and figures presented … illuminate the worldwide neglect of a major cause of public ill-health and reveal the inadequacies of responses to it in countries throughout the world” [2]. In addition, and standing as a barrier to improvement, it is not certain that there is a universal view of what optimal headache services should look like, or, indeed, of the meaning in this context of “optimal”.

It is axiomatic that health-care systems should aspire to high quality of care, but progress toward this requires that quality can be assessed. Before this is possible, “quality” must first be defined. Donabedian [3], in a view now widely endorsed, suggested that quality of care should be considered in each of three domains: “structure” (the attributes of the settings in which care occurs); “process” (the giving and receiving of care); and “outcome” (the effects that care has on health status). Donabedian [4] also described seven attributes that in his view collectively defined health-care quality: efficacy, effectiveness, efficiency, optimality, acceptability, legitimacy and equity. A definition of quality of health care offered by the US Institute of Medicine (IOM) put the emphasis on outcomes: the degree to which health-care services for individuals and populations increase the likelihood of desired health outcomes and are consistent with current professional knowledge [5]. The IOM specified six attributes of quality, differing somewhat from Donabedian’s seven: safety, timeliness, effectiveness, efficiency, equity and patient/family-centerdness.

What are needed for services delivering headache care are agreed and widely accepted quality measures. Their application to existing services might, by establishing standards of care, providing benchmarks and revealing deficiencies, motivate and guide improvements. Their employment will bring clarity of purpose to initiatives aimed at developing services where none exist. As a project within the Global Campaign against Headache [6, 7], which seeks both to develop and improve services, we performed a systematic literature review with the aim of identifying and summarizing existing information on quality indicators for headache care. Specifically, our questions were: did indicators exist, how had they been developed, what aspects of headache care did they relate to and how and with what utility were they being used?

## Methods

### Search strategy

During 2009 we searched Medline, EMBASE and CINAHL without language restrictions for relevant articles using the search terms “headache disorders” and “health care quality” for both title and text words. We combined the individual search results using the Boolean operator “AND”. We extended the search to articles containing text words “headache care*.tw” or “headache service*.tw” in order to ensure identification of all relevant articles. The thesaurus terms and text words used for these searches are shown in Table 1. The search was limited to articles from 1988 onwards, this being the year in which universally accepted definitions of headache disorders were first published [8]. We updated the search in February 2012 and found no new articles specifically related to the definition of quality or to quality indicators in the context of headache care. All identified articles were transferred to Reference Manager^®^, a bibliographic software program.

**Table 1:** Search terms in the literature review

	MEDLINE	EMBASE	CINAHL
Thesaurus terms for ‘headache’	Headache disorders/ Headache disorders primary/ Migraine disorders/ Migraine with aura/ Migraine without aura/ Tension type headache/	Headache/ Primary headache/ Migraine/ Tension headache/ Chronic daily headache/	Headache/ Migraine/ Tension headache/
Text words for ‘headache’ (Title & Abstract)	Chronic daily headache*.tw. Migraine*.tw. Tension headache*.tw. Tension type headache*.tw. TTH*.tw. CDH*.tw.	Chronic daily headache*.tw. Migraine*.tw. Tension headache*.tw. Tension type headache*.tw. TTH*.tw. CDH*.tw.	Chronic daily headache* Migraine* Tension headache* Tension type headache* TTH* CDH*
Thesaurus terms for ‘health care quality’	Quality of health care/ Quality assurance health care/ Total quality management/ Outcome and process assessment (health care)/ Quality indicators, health care/ Peer review, health care/ Programme evaluation/ Bench marking/ Clinical audit/ Medical audit/ Nursing audit/	Health care quality/ Quality indicators, health care/ Peer review, health care/ Medical audit/	Quality assessment/ Clinical Indicators/ Nursing audit/ Outcome assessment/ Outcome assessment information set/ Peer review/ Process assessment (health care)/ Program evaluation/ Quality of health Care/ Quality of nursing care/ Quality assurance/ Quality improvement/ Evaluation and quality improvement program/ Benchmarking/ Quality management organizational/
Text words for ‘healthcare quality’ (Title & Abstract)	Health service* research*.tw. Health service* evaluation*.tw.	Continuous quality improvement*.tw. Health service* research*.tw. Health service* evaluation*.tw. Nursing audit*.tw.	Continuous quality improvement* Health service* research* Health service* evaluation*

#### Inclusion and exclusion criteria

**Fig. 1:**
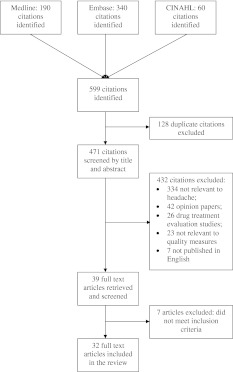
Flow of studies through the review

Two of the authors (SP and MP) screened titles and abstracts for relevance using predefined inclusion and exclusion criteria. Studies were included if they reported the development or application of quality indicators for headache care or evaluated other aspects of service provision for headache disorders. Articles unrelated to primary headache disorders, review articles, drug treatment studies, case reports, letters and comments, and papers that were not written in English were excluded. We obtained full-text versions of papers selected for further review, and again evaluated these against our inclusion and exclusion criteria. Any disagreements were resolved by consensus. Figure 1 shows the flow of studies through the review.

### Data extraction and analysis

A data extraction form was developed to summarize the relevant results of the studies selected. Formal statistical analysis was not possible because of the heterogeneity of evidence and measures in the selected articles, so a narrative synthesis of the findings was prepared.

## Results

A total of 32 studies met criteria for inclusion in the review. Four [9–12] reported development of quality indicators for use in headache-service delivery; 28 [13–40] (listed in Table 2) evaluated aspects of headache care quality in a variety of settings, but did not employ formal quality measures to do so.

**Table 2:** Studies (*n* = 28) (identified by first author) evaluating headache care components associated with quality

Author	Process				Outcome		
	Diagnosis	Treatment	Management	Services	Clinical symptoms	Impact	Satisfaction
Agostoni [13]	Diagnostic accuracy						
Belam [14]						Impact Quality of life	
Bigal [15]	Diagnostic rates Investigations						
Blumenfeld [16]		Prophylactic	Patient education	Consultations		Quality of life	Physicians’ satisfaction
Blumenthal [17]	Diagnostic accuracy	Acute			Headache severity		
Campinha-Bacote [18]			Patient education Recognition of triggers		Headache severity Headache frequency	Economic burden Work productivity	Patient satisfaction
Clarke [19]	Diagnostic accuracy Investigations						
Clarke [20]	Diagnostic accuracy Investigations	Treatment generally			Headache improvement or deterioration		
Davies [22]	Investigations				Pain severity		Patient satisfaction
Dowson [22]	Diagnostic rates Investigations	Acute Prophylactic					
Elsner [23]	Diagnostic rates		Physician education		Pain severity		
Gahir [24]				Consultations (GP and pharmacist)			
Gahir [25]			Consultations (emergency department)				
Harpole [26]		Acute Prophylactic		Referrals		Disability Quality of life	Patient satisfaction
Harpole [27]		Prophylaxis		Consultations (emergency department)		Disability	
Karli [28]	Diagnostic accuracy	Acute Prophylaxis	Physician education				
Larner [29]		Prescription rates		Consultations (GP)			
Latinovic [30]		Prescription rates		Consultations (GP) Referrals			
Magnusson [31]					Headache frequency Headache severity	Disability Quality of life	
Maizels [32]	Diagnostic rates	Prescription rates Acute Prophylaxis OTC use		Consultations (emergency department)			
Maizels [33]	Diagnostic rates	Acute medication Prescription rates					
Matchar [34]			Patient education			Disability Quality of life Depression	
Melchart [35]	Diagnostic rates	Medication use			Pain intensity Pain frequency	Functional ability Quality of life Health-related behavior	
Offredy [36]							Patient satisfaction
Ridsdale [37]				Cost of service			Patient satisfaction
Soon [38]	Diagnostic rates	Acute medication Prophylactic medication				Disability Quality of life	
Vincent [39]	Diagnostic accuracy	Prophylactic medication					
Zeeberg [40]	Diagnostic rates	Acute medication Prophylactic medication		Referrals	Headache frequency		

The quality domain ‘structure’ is not represented in the table as none of the articles addressed this domain

*OTC* over-the-counter medication

### Development of quality indicators

Tables 3 and 4 list the characteristics and results of the four studies that reported on the development of a total of 55 quality indicators. These indicators are categorized according to domain of application: structure, process or outcome of headache care. Within these domains are sub-domains into which they are further categorized. In fact, none of the studies developed indicators of quality of structural aspects of headache care. All, however, selected indicators of process, and 36 of the 37 process measures evaluated diagnostic or treatment procedures (for example, the use of specific diagnostic procedures when certain features were present in history or physical examination, or the prescription of specific medications for certain types of headache). Such process indicators commonly use “if–then” statements, and therefore do not apply to all patients evaluated for headache. Only two studies developed indicators of outcome of headache care: a total of 18 indicators, focusing on frequency and severity of headaches or on uptake of care. None of these outcome indicators evaluated disability, quality of life or patients’ satisfaction with care.

**Table 3:** Quality domains for which indicators were developed within the four studies

Quality domain	Sub-domain	Indicators developed (*n*)			
		McGlynn et al. [10]	Marshall et al. [9]	Leas et al. [12]	Ferrari et al. [11]
Structure		0	0	0	0
Process	Diagnosis	13	0	5	0
	Treatment	8	4	6	0
	Referral for care	0	1	0	0
Outcome	Headache severity and frequency	0	0	0	6
	Disability	0	0	0	0
	Quality of life	0	0	0	0
	Satisfaction with care	0	0	0	0
	Uptake of care	0	0	9	3

**Table 4:** Existing headache quality indicators (developed within the four studies)

Domain	Sub-domain	Quality indicator(s)
McGlynn et al. [10]		
Diagnosis	History-taking	Patients with new onset headache should be asked about:
		(1) the location of the pain (2) their associated symptoms (3) their temporal profile (4) the degree of severity of the headache (5) family history of headache (6) any possible aggravating or alleviating factors
	Physical examination	Patients with new onset headache should have an examination evaluating: (1) the cranial nerves (2) the fundi (3) deep tendon reflexes (4) their blood pressure
	Investigations	(1) CT or MRI scanning is indicated in patients with new onset headache and an abnormal neurological examination (2) CT or MRI scanning is indicated in patients with new onset headache and severe headache (3) Skull X-rays should not be part of an evaluation for headache
Treatment	Acute	(1) Patients with acute mild migraine or tension headache should have tried aspirin, Tylenol, or other nonsteroidal anti-inflammatory agents before being offered any other medication (2) For patients with acute moderate or severe migraine headache, one of the following should have been tried before any other agent is offered: ketorolac, sumatriptan, dihydroergotamine, ergotamine, chlorpromazine, or metoclopramide (3) Recurrent moderate or severe tension headache should be treated with a trial of tricyclic antidepressant agents, if there are no medical contraindications to use (4) Sumatriptan and ergotamine should not be concurrently administered (5) Opioid agonists and barbiturates should not be first-line therapy for migraine or tension headaches (6) Sumatriptan and ergotamine should not be given in patients with a history of uncontrolled hypertension (7) Sumatriptan and ergotamine should not be given in patients with a history of ischemic heart disease or angina
	Prophylactic	(1) If patients have more than two moderate to severe migraine headache each month, then prophylactic treatment with one of the following agents should be offered: β-blockers, calcium channel blockers, tricyclic antidepressants, naproxen, aspirin, fluoxetine, valproate, or cyproheptadine
Referral		None
Outcome		None
Marshall et al. [9]		
Diagnosis		None
Treatment	Acute	(1) Sumatriptan should not be prescribed for migraine in patients with angina
	Prophylactic	(1) Prophylaxis treatment should be offered in patients with severe and disabling migraine (2) The following agents should be prescribed as first line for prophylaxis of migraine unless contraindicated; beta blockers, tricyclic antidepressants, pizotifen (3) Beta blockers should not be prescribed for migraine in patients with asthma
Referral		(1) Patients should be referred urgently for specialist care and investigation if the presenting headache is accompanied by; suspected raised intracranial pressure, new onset seizure, focal neurological signs or papilloedema
Outcome		None
Leas et al. [12]		
Diagnosis	Investigations	% of patients who had (1) a computerized tomography scan (2) a magnetic resonance imaging scan
	Other	% of patients (1) who had a diagnosis of migraine (2) who had a diagnosis of headache not otherwise specified (iii) with a prescription for triptan who have a diagnosis of headache not otherwise specified
Treatment	Acute	% of patients who had a prescription for (1) a triptan (2) an ergot alkaloid/derivative
	Prophylactic	% of patients (1) who had a prescription for a migraine preventive (2) overusing triptans who have a prescription for migraine preventative
	Other	% patients who had (1) a prescription for a triptan and a migraine preventative (2) triptan overuse
Referral		None
Outcome	Uptake of care	% of patients (1) with at least 1 migraine-related emergency department visit who had a follow-up visit (2) who had a primary-care physician visit for migraine (primary diagnosis) (3) who had a primary-care physician visit for migraine (any diagnosis) (4) who had a specialist visit for migraine (5) who had an emergency department visit for migraine (6) who had an acute hospitalization for migraine
		and
		Number of (7) emergency department visits (8) acute hospitalizations (9) acute inpatient days
Ferrari et al. [11]		
Diagnosis		None
Treatment		None
Referral		None
Outcome	Headache severity and frequency	(1) % of chronic headache sufferers who reported a decrease of at least 50 % in headache frequency at discharge from day hospital or ordinary hospital (2) % of chronic headache sufferers overusing drugs who upon discharge from day hospital or ordinary hospital after detoxifying therapy, reduce their intake of analgesics by at least 50 % (3) % of patients re-admitted to day hospital or ordinary hospital within 28 days of discharge (4) % of patients referred by their general practitioner for a clinical examination within 28 days of discharge (5) % of patients returning after discharge with side effects due to treatment prescribed (6) % of patients returning after discharge owing to inefficacy of treatment prescribed
	Uptake of care	(1) % of patients with an appointment who do not turn up for their first clinical examination (2) % of patients with an appointment who do not turn up for their examination to complete the diagnostic picture (3) No. of phone calls, fax messages, emails from general practitioners to the headache center

*CT* X-ray computerized tomography, *MRI* magnetic resonance imaging

All four studies were conducted in developed western countries, but they varied in their goals and in their methods of indicator development. Two [9, 10] aimed to evaluate overall quality of care provided at a national level for a number of common conditions, headache among them. They employed elaborate consensus methods for the development of indicators focused on process, since the scope of the projects made it unfeasible to collect outcome information. The other two studies [11, 12] had the more specific goal of evaluating migraine care, either within health plans or within a specialty clinic. They employed less extensive, more practical methods to develop quality indicators, but concentrated on treatment outcomes. These four studies are the key, and we describe them below in more detail.

McGlynn et al. [10] developed quality indicators for 30 acute and chronic conditions; the purpose was to assess overall quality of medical care provided to adults in the United States (US). Marshall et al. [9] developed quality indicators for 18 diseases in a project to assess quality of health care delivered to adults in primary care in the United Kingdom (UK). In both studies, the process of developing quality indicators began with a literature review. McGlynn et al. [10] then used a modified Delphi method to develop a final set of indicators. US experts in each disease area rated putative quality indicators on a 9-point scale from 1 (“not valid”) to 9 (“very valid”). Only those with a median composite score of ≥7 were selected. Similar methods were employed by Marshall et al. [9]. UK primary-care experts rated putative indicators on a 9-point validity scale, and also rated the necessity of including such information in patient records. Only those that achieved mean scores of ≥8 for validity and ≥6 for necessity scale were selected.

Leas et al. [12] developed a set of 20 quality of care measures specific to migraine—the only set of indicators to include both process and outcome measures. Existing measures were identified through a literature review and other candidate measures through telephone interviews with leaders in migraine care, health-care purchasing and managed care. An advisory board of experts then discussed all putative measures and, by consensus, selected a final set of indicators for testing. In contrast to those developed by McGlynn and by Marshall, this set of quality indicators included a number that evaluated the costs and outcomes of treatment.

Ferrari et al. [11] developed a set of quality indicators for headache care in conjunction with the staff of a specialist headache center and a quality assurance office. The goal of these indicators was to ensure the provision of consistent, high-quality care within a specialized headache treatment center. This set of indicators did not include any measures of headache care structure or process but instead focused exclusively on specific aspects of treatment outcome.

### Evaluation of specific aspects of headache care

Table 2 lists the 28 studies [13–40] that assessed specific components of headache care associated with quality, without employing formal quality indicators. It also indicates the domains of quality (and sub-domains) addressed by each. All these studies were conducted in highly resourced locations, but evaluated strategies and aspects of care delivered in a range of settings by different practitioners. Four studies [18, 23, 28, 30] were conducted in primary care, three [14, 36, 37] in intermediate care, where treatment was provided by general practitioners with a special interest in headache, and one [20] in a setting where specialist nurses provided care. Six studies [21, 22, 31, 38–40] were conducted in specialty headache clinics, one [35] in an inpatient treatment setting, and five [13, 15, 17, 32, 33] evaluated headache care in emergency departments. Four studies [17, 19, 28, 39] assessed diagnostic accuracy, while eight [16, 18, 23, 26, 27, 31, 34, 35] evaluated the effects of specific strategies of headache management. Only two of the studies [31, 34] included a control group; one of these [34] was a randomized clinical trial. Some of the studies evaluated outcomes using existing general or headache-specific disability or quality of life instruments. Table 5 (available on-line) documents the study characteristics in detail.

We found no studies that reported on the reliability, validity, practicality or implementation of any of the 55 indicators.

## Discussion

While many quality indicators have been developed to evaluate headache care, evidence regarding their reliability, validity and practicality is lacking. They emphasize processes of care rather than outcomes, and ignore structure. Most cover areas of routine assessment, but do not clearly specify the tool or process to be used in evaluation. Others describe desirable treatment in broad terms, including diagnosis, management or administration of particular tests or drugs. None of the identified measures report inter-rater reliability or other psychometric properties. They are not clearly applicable to different levels and locations of headache care. There is no evidence that any of them have been used for quality improvement, although this is presumably the purpose for which they were developed.

The process of developing quality indicators was not, in any of the studies, begun with, or therefore informed by, an agreed definition of “quality”. What is surprising is that neither did these studies attempt to construct a definition, in the specific context of headache care, as a prerequisite for developing indicators of it. While quality is important in health care for any condition, and may to that extent have a general definition, there are aspects of it that are specific to or of particular importance in headache care. Furthermore, it is not clear that a universally accepted general definition of quality of care does exist; even its attributes are not wholly agreed [4]. At issue here is whose perspectives matter in the meaning and assessment of health-care quality: patients’, health-care providers’ or payers’? Assuming they all do to an extent, and they are not perfectly aligned, which have priority? Quality is not necessarily coupled to financing: there is no direct relationship between better outcomes and the amount spent on health care [41]. Improving the quality of care for headache disorders goes beyond better diagnosis and good treatment, since large numbers of people with headache do not consult doctors and hence will not benefit from improvements in care processes. There is clear evidence of high barriers to care [2], and the need to dismantle them is high on the agenda for headache-service quality improvement. Sorting out these issues appears to be a prerequisite for developing quality indicators for headache services, but it has not been done.

Our study has strengths: the systematic nature of the literature search and review and the incorporation of information from studies that provided indirect evidence relevant to the development of quality indicators. Its main limitation was that we were able to search only for publicly available quality indicators and implementation studies: it is possible that insurance companies or other health management organizations have developed, validated and implemented proprietary quality indicators that have not been published. Of course, if such indicators exist, it might be asked whose perspective(s) they reflect.

In conclusion, we identified a number of studies providing evidence of the value of specific types, strategies and measures of headache treatment, but much further work is needed to incorporate these findings into the development of valid and practical quality indicators. There is no agreed definition of “quality” of headache care, and no considered view on how the non-aligned perspectives of different stakeholders in headache care should be placed in order of priority. Consensus on these issues is urgently required if health care for headache—clearly suboptimal throughout the world—is to be improved. This is a priority for patients and for public health.

## Electronic supplementary material

Below is the link to the electronic supplementary material.

Supplementary material 1 (PDF 200 kb)
