# Supplementary material for: Quality in the provision of headache care. 1: systematic review of the literature and commentary
Source: J Headache Pain. 2012 Jun 27;13(6):437–47. doi: 10.1007/s10194-012-0466-1 (PMC3464474; doi:10.1007/s10194-012-0466-1)
Supplement: Supplementary file 1 — Supplementary material 1 (PDF 200 kb) [file 10194_2012_466_MOESM1_ESM.pdf]

Quality in the provision of headache care. 1. Systematic review of the literature, and commentary

Michele Peters (University of Oxford, michele.peters@dph.ox.ac.uk), Suraj Perera, Elizabeth Loder, Crispin Jenkinson, Raquel Gil Gouveia, Rigmor Jensen, Zaza Katsarava and Timothy J Steiner

Journal of Headache and Pain

Table 5: Full details of studies (n-28) evaluating headache care components associated with quality

Author	Population and setting	Study aim	Methodology	Authors' principal findings/conclusions
Agostini *et al*, 2004 [13]	346 patients attending an Italian ED with headache as their major complaint	To evaluate reliability of headache diagnosis made by different physicians in ED and validity of diagnosis comparing ED physicians and neurologists with diagnosis made by a headache expert	Over 18 months, every patient attending ED for headache was independently diagnose by an ED physician, an ED neurologist and a headache specialist. The diagnosis made by headache specialist was based on IHS criteria.	Of 346 patients, only 117 (33.8%) examined by the three different physicians. ED physician diagnosed 62.4% with idiopathic headaches compared to 71.8% by ED neurologist. Symptomatic headache diagnosed in 25.6% by ED physician and 29.1% by ED neurologist. Headache of uncertain origin diagnosed in 8.5% by ED physician and 2.6% by ED neurologist. Agreement in diagnosis was fair between ED physician and headache specialist (kappa=0.40) and moderate for the other two pairs (kappa=0.57 and 0.60). Agreement was moderate in patients with a first episode of headache between ED physician and ED neurologist (kappa=0.58) and fair between ED neurologist and headache specialist (kappa=0.24). Agreement was moderate in patients with complete impairment of daily living activities between ED physician and headache expert (kappa=0.51) and substantial for the other two pairs (kappa=0.65). Highest level of agreement was in a small number (n not stated) of patients arriving to ED by ambulance (kappa=0.78). Overall, agreement was highest between ED neurologist and headache specialist, and lowest between ED physician and headache specialist.<br><br>The authors concluded that patients seen by ED physician can be managed fairly successfully, except those presenting with a first headache episode, who need to be seen by a neurologist.
Belam *et al*, 2005 [14]	8 migraineurs attending an intermediate care UK headache clinic	To explore the experience of suffering from migraine in order to inform health service delivery	Qualitative interview study with migraine patients	The main themes that emerged were 'impact on life (everyone is different)', 'making sense of the problem', 'putting up with it' and 'doing something about it'. The authors concluded that, despite a significant impact on quality of life, patients' needs remained largely unmet.

Michele Peters (University of Oxford, michele.peters@dph.ox.ac.uk), Suraj Perera, Elizabeth Loder, Crispin Jenkinson, Raquel Gil Gouveia, Rigmor Jensen, Zaza Katsarava and Timothy J Steiner

Journal of Headache and Pain

Table 5 (contd): Full details of studies (n-28) evaluating headache care components associated with quality

Author	Population and setting	Study aim	Methodology	Authors' principal findings/conclusions
Bigal *et al*, 2000 [15]	1,254 patients referred from primary care to a Brazilian ED for headaches refractory to treatment or query about the diagnosis	To evaluate quality of primary care for acute headaches	Retrospective review of records of patients who have consulted the ED over a 1-year period.	Most patients (94.9%) spent <12 hours in ED (*i.e.*, non-hospitalized patients); 64 (5.1%) were hospitalized. Primary headaches were most common (77.0%) in non-hospitalized patients. Other headaches included those secondary to neurological disorders (n=15, 9.1%) or to systemic disorders (n=23, 13.9%). Headaches secondary to neurological disorders were most common among hospitalized patients (n=33, 51.5%). A higher proportion of non-hospitalized than of hospitalized patients were diagnosed with migraine (n=93, 56.4% *vs.* n=14, 21.9%) or TTH (n=25, 15.1% *vs.* n=2, 3.1%). In non-hospitalized patients, diagnosis was predominantly clinical examination with <10% having CT and 1.2% spinal taps. In contrast, 56.3% of hospitalized patients had CT or MRI and 39% spinal taps.<br><br>The authors concluded that headache care by primary care is unsatisfactory as many patients are referred to tertiary care services.

Michele Peters (University of Oxford, michele.peters@dph.ox.ac.uk), Suraj Perera, Elizabeth Loder, Crispin Jenkinson, Raquel Gil Gouveia, Rigmor Jensen, Zaza Katsarava and Timothy J Steiner

Journal of Headache and Pain

Table 5 (contd): Full details of studies (n-28) evaluating headache care components associated with quality

Author	Population and setting	Study aim	Methodology	Authors' principal findings/conclusions
Blumenfeld & Tischio, 2003 [16]	497 adult patients with primary headache disorders referred to a headache care programme by their primary care provider.	To assess effectiveness of a disease management model for primary headache by evaluating patients' quality of life, headache-related visits to primary care and ED, and patients' and physicians' satisfaction.	Prospective cohort pilot study in adults with primary headaches. Patients attended educational session by neurologist and nurse practitioner. Subsequently, nurse practitioner evaluated patient and developed a treatment plan. Patients completed Migraine-Specific Quality of Life (MSQL) and SF-36 questionnaires at baseline, 8-week follow-up visit and after 6 months. Data on provider visits, medication use and diagnostic testing drawn from patients' medical records. Patients' satisfaction assessed at 8-weeks by single question on headache improvement. Physicians' satisfaction assessed by questionnaire sent at end of study.	422 of 497 attended 8-week follow up visit and 12 completed 8-week questionnaire by telephone. The remaining 63 patients could not be followed-up. At 8-week follow up, 92% of patients reported improvement in their headaches, 7% were unchanged and 1% felt worse. Quality of life was significantly improved on all 8 SF-36 dimensions ($p<0.001$) for 356 patients who completed questionnaire at baseline and for one or more follow-up periods. Greatest improvement was measured between baseline and 8-week follow up, with improvements levelling off at 6 months for most dimensions. All dimension scores, apart for Physical Functioning and General Health, improved by 5 points or more at 8-week follow up, indicating clinically significant improvement. 363 patients completed MSQL questionnaire at baseline and for one or more follow-up periods. There was significant improvement ($p<0.001$) in mean scores for all three dimensions at 8 weeks and 6 months. Numbers for reasons of visits (there could have been more than one type of visit) for 'headache' (296 visits pre- *vs* 122 post-programme) and 'headache and other' (147 pre- *vs* 56 post-programme) decreased significantly ($p<0.001$). There was no significant difference for non-headache visits. Numbers for locations of visits (this could be more than one location per patient) decreased significantly in primary care (411 pre- *vs* 267 post programme, $p<0.01$) and ED (81 pre- *vs* 53 post programme, $p<0.01$), whilst number of visits to a neurologist significantly increased (8 pre- *vs* 30 post-programme, $p<0.01$). The use of injected and oral narcotics decreased significantly ($p<0.01$) from pre- to post-programme, whilst use of triptans increased slightly but not significantly. 86% of 77 primary care physicians were satisfied with the programme overall, about 10% were neutral and <4% were somewhat dissatisfied. The authors concluded that this type of disease management model leads to improvements in individualized patient care and empowered patients to take control of their care through shared decision making.

Michele Peters (University of Oxford, michele.peters@dph.ox.ac.uk), Suraj Perera, Elizabeth Loder, Crispin Jenkinson, Raquel Gil Gouveia, Rigmor Jensen, Zaza Katsarava and Timothy J Steiner

Journal of Headache and Pain

Table 5 (contd): Full details of studies (n-28) evaluating headache care components associated with quality

Author	Population and setting	Study aim	Methodology	Authors' principal findings/conclusions
Blumenthal *et al*, 2003 [17]	57 patients attending US ED with headache	To assess ED physicians' diagnosis and treatment for primary headache and assess patients' responses to headache treatment in ED.	Observational study with diagnostic accuracy assessed by comparing diagnoses made from a patient self-reported questionnaire based on IHS criteria to diagnoses of ED physicians. Patients also completed questionnaire on response to treatment (in ED and at 24 hours post treatment).	Most patients (95%) met IHS criteria for migraine but ED physicians diagnosed only 32%; the majority (59%) were diagnosed with 'cephalgia' or 'headache not otherwise specified'. Respondents had taken a mean of 3 (range 1-9) non-prescription medications for their headaches and 82% had previously taken a mean of 3.4 (range 1-13) prescription medications. 49% had never taken a triptan but 21% had taken a triptan on the day they came to ED. 95% had previously seen an average of 2.9 (range 1-10) different physicians. Only 4 patients received migraine-specific acute treatment (3 a triptan and 1 dihydoergotamine), whilst 65% were treated with a 'migraine cocktail' of NSAID, dopamine antagonist and antihistamine. 24% were given an opioid with or without antinauseant and 4 were given no medication. ED physicians reported complete headache relief in 35% of patients and 'headache improvement' in 29% at time of discharge. One patient was reported unchanged and in 34% no comment on headache response was made in the chart. 42% of patients reported excellent headache relief on discharge and 10% poor relief. 28% of patients reported completed headache relief 2 hours after treatment and 36% complete relief after 12 hours. 60% of patients still had headache 24 hours after discharge from ED.

Table 5 (contd): Full details of studies (n-28) evaluating headache care components associated with quality

Author	Population and setting	Study aim	Methodology	Authors' principal findings/conclusions
Campinha-Bacote *et al*, 2005 [18]	2,134 migraine patients and their primary care physicians in the US	To evaluate effectiveness of a migraine disease management programme.	Prospective migraine management programme (MMP) which delivered educational material (newsletters and other written materials) by mail both to physicians and to patients. Aims were to improve patient satisfaction with migraine care, decrease migraine frequency, severity and related disability, improve effectiveness of migraine treatment and work productivity, improve physician's diagnosis and treatment of migraine and health care resource utilization.<br><br>Patients and their physicians studied before- and after-intervention. Patients completed Migraine Therapy Assessment Questionnaire (MTAQ) at baseline and follow up.	Of 2,134 patients completing baseline questionnaire, only 789 completed follow up questionnaire. In these there was significant improvement in symptom relief within 2 hours, satisfaction with migraine treatment, understanding triggers. Patients reported decreases in migraine frequency, missed days of work/school and ED visits. Poor symptom control decreased from 46.5% to 41.3% (p=0.0036), high attack frequency from 35.6% to 31.4% (p=0.02), knowledge/behavior barrier from 78.3% to 72.7% (p=0.0016), economic burden from 65.3% to 53.7% (p<0.0001) and dissatisfaction with treatment from 38.8% to 31.0% (p<0.001). Improvements were greatest for economic burden and dissatisfaction with treatment (reduced by about 20%). Men reported significant improvements in recognizing triggers, satisfaction with treatment and decrease in missed activities, whilst women reported improvements in time to alleviation of symptoms, ability to return to normal activities, recognizing migraine triggers and satisfaction with migraine therapy, and decreases in migraine frequency, missed activities and use of ED. In women who completed both baseline and follow-up questionnaires, poor symptom control decreased from 46.0% to 39.8% (p=0.0014), knowledge/behavior barrier from 76.3% to 71.4% (p=0.0142), economic burden from 66.1% to 54.9% (p<0.001) and treatment dissatisfaction from 37.1% to 29.7% (p=0.0003). In men, knowledge/behavior barrier decreased from 88.4% to 79.0% (p=0.0193), economic burden from 60.6% to 47.7% (p=0.0055) and dissatisfaction with treatment from 47.3% to 37.6% (p=0.0118). Patients who returned only baseline questionnaire were more likely than patients who also completed follow up questionnaire to report poorer symptom control (57.8% *vs* 46.9%, p<0.0001), greater economic burden (67.9% *vs* 63.5%) and dissatisfaction with treatment (52.5% *vs* 40.0% p<0.0001). The satisfaction survey revealed that only 33% of patients who had been recommended to contact a doctor actually did so.

Table 5 (contd): Full details of studies (n-28) evaluating headache care components associated with quality

Author	Population and setting	Study aim	Methodology	Authors' principal findings/conclusions
Clarke *et al*, 2005 [19]	239 headache patients and 13 professional role players presenting with headache in UK	To compare diagnostic abilities between a specialist nurse and a consultant neurologist	Non-acute patients who had consulted a neurologist over 6 months were invited to attend a headache clinic. Additionally, role players were trained to mimic sinister headaches. Patients diagnosed by both neurologist and nurse independently (in random order) according to IHS criteria. Alternatives to primary (most severe) diagnosis (*ie*, secondary and tertiary diagnoses) permitted to allow for uncertainty.	According to neurologist, most common diagnoses were TTH headache (47%) and migraine (39%). Exact agreement between neurologist and nurse in 68% of patients with TTH, 77% with migraine and 34% with other types of headache (when considering nurse's primary diagnosis only). When comparing neurologist diagnosis to all three of nurse's diagnoses (primary to tertiary), agreement for TTH was 92%, migraine 91% and other types of headache 61%. In 30 patients where the nurse had recorded none of the neurologist's diagnoses, most were misclassifications between TTH and migraine. The nurse referred more participants for further medical opinion (58%) than the neurologist (37%). 82 patients had 108 tests. Of 49 CT scans, 44 were normal, 4 showed some clinically irrelevant abnormalities and one identified a suspected tumour. Of 20 MRIs, 12 were normal and 8 had clinically irrelevant abnormalities. Patients' records were checked after 6 months to look for missed sinister pathologies: none were discovered. Both nurse and neurologist misdiagnosed the same 3 of 13 role players.

Table 5 (contd): Full details of studies (n-28) evaluating headache care components associated with quality

Author	Population and setting	Study aim	Methodology	Authors' principal findings/conclusions
Clarke *et al*, 2008 [20]	1,347 patients with headache newly referred to a nurse-led headache services in UK	To evaluate a nurse-led headache service over 2 years, using IHS criteria for diagnosis and British Association for the Study of Headache guidelines for treatment.	2 year prospective study to monitor patient demographics, diagnoses and investigations. Patients rated their responses to treatment since last appointment on 7-point Likert scale. Medications and doses recorded at each visit. Waiting and referral times assessed retrospectively.	Most common diagnoses were migraine (47%) and TTH (41%). Investigations were required by 17% of patients compared to 37% from consultant-led service. Follow-ups were planned for 63% (n=836) of patients, 77% of whom were invited for second review. Of 278 (first) follow-up patients with migraine, 34% (n=95) reported no change. Of the others, more (50%) reported improvement rather than deterioration (16%) (p<0.001). 48% of TTH patients reported no change; of the others, more (48%) reported improvement than deterioration (4%) (p<0.001). 182 patients attended the second follow-up. 34 of 107 (32%) migraine patients reported no change; of the remainder, more (47%) reported improvement than deterioration (22%) (p=0.002). 22 of 45 (49%) TTH patients reported no change; 33% reported improvement and 18% deterioration (NS). Treatment for 107 migraine patients who attended 2 follow-ups closely followed protocol, with steady increase in more 'aggressive' treatments such as triptans. TTH treatment also followed protocol. Waiting times over 13 weeks were eliminated and total number of patients waiting to see a neurologist decreased.<br><br>**Note**: Nurse service not, in most part, compared to a standard. Implied that fewer investigations is positive. Data available for minority of patients initially referred. Unclear how many followed up at which times, and unknown whether there were differences between those invited for follow-up and those not, or between those who attended and those who did not.

Table 5 (contd): Full details of studies (n-28) evaluating headache care components associated with quality

Author	Population and setting	Study aim	Methodology	Authors' principal findings/conclusions
Davies, Glynn and Kadry, 2003 [22]	52 patients, most with daily or near-constant headaches (CDH) in UK	To assess patients' expectations when referred to a combined headache clinic (consultant neurologist specializing in headache plus consultant anaesthetist)	Retrospective chart review over 2-year period. Patients administered a questionnaire about pain and psychological characteristics prior to consultation, and asked about their expectations during consultation.	Patients had suffered from headache for mean of 8.25 (range 1-40) years. 40 of 52 (77%) were still concerned about cause of their headaches and 13 (33%) of these wanted further investigations. 35 of 52 (67%) were more concerned with finding cause than with receiving symptomatic treatment; 25 of these (71%) were reassured by addressing unrealistic expectations. 14 of 52 (27%) were discharged (9 satisfied and accepting treatment, 5 unsatisfied and continuing to look for a cause of their headaches). 5 of 52 (10%) demanded further investigations and did not attend follow up appointment. 33 of 52 (64%) accepted symptomatic management within the clinic.
Dowson, 2003 [22]	458 patients who had attended a specialty headache clinic in the UK	To examine profile of patients attending a specialist headache clinic, to detect changes in management and to evaluate primary care referral patterns	Retrospective audit of a 3-year period to evaluate diagnoses given, medications history and investigations conducted.	Most (276, 60%) patients diagnosed with CDH, 153 (33%) with migraine, 19 (4%) with 'short sharp headaches' and 24 (5%) with cluster headache. Some patients diagnosed with >1 type of headache. More women consulted for CDH and migraine; cluster headache was diagnosed in men only. Proportion of patients consulting for CDH was relatively constant over 3 years, whereas proportion consulting for migraine fell from 37% to 29%. Prior to attending clinic, 183 (66%) of CDH patients relied on analgesics and only 42 (15%) used prophylactics. Most CDH patients (204, 74%) were prescribed prophylactic medication by the specialist. Migraine patients predominantly (89, 58%) relied on analgesics and 44 (29%) on triptans. Use of prophylaxis (19, 12%) and of ergots (11, 7%) for migraine were both low. The proportion of prescriptions for triptans increased slightly from 26% to 32% over the 3 years. The specialist recommended analgesics for 14 (9%) patients, triptans for 124 (81%) and prophylaxis for 130 (85%). Investigations were conducted in19 (9%) patients in the first year, 17 (10%) in the second and 17 (22%) in the third. Most investigations were for CDH (58%) or migraine (11%).

Table 5 (contd): Full details of studies (n-28) evaluating headache care components associated with quality

Author	Population and setting	Study aim	Methodology	Authors' principal findings/conclusions
Elsner *et al*, 2004 [23]	Headache patients in general practice in Germany	To describe patient outcomes after standard pain management and after delivery of an intervention for physicians.	Observational study of 2 strategies of headache care: outpatient pain management provided by GP (1 year period) (Phase 1) OR pain management by GP after educational programme from pain specialist (2-year period) (Phase 2). Data on diagnosis and treatment collected from patients and GPs by questionnaire at T1 (baseline), T2 (after 4-6 weeks) and T3 (10-12 weeks). Patients also completed Brief Pain Inventory (BPI), Perception of Pain Scale (PPS), Symptom Checklist (SCL-90-R), SF-36 and Client-Satisfaction Questionnaire-8.	80 physicians participated: yearly questionnaires on attitudes and knowledge returned only by 30. Patients' demographics did not differ between phases 1 and 2. Due to high number of dropouts, variables only compared at T1 and T2. Pain intensity on BPI decreased from T1 to T2 ($p<0.001$) as did pain-related impairment ($p<0.005$). Affective descriptors on PPS decreased significantly by T2 ($p<0.005$) but evaluative descriptors did not. In both phases, SF36 scores were lower than the norm; changes between T1 and T2 were significant for physical component score ($p=0.008$) but overall changes in SF-36 were minimal. Changes in SCL-90-R scores were significant ($p<0.05$) but did not differ between phases 1 and phase 2. At the end of phase 2, more GPs reported knowing headache guidelines and headaches diaries, triptans and anti-depressants were used more frequently. Migraine and TTH were most frequent diagnoses; cervicogenic headache was diagnosed disproportionally often in Phase 1 (33%) but significantly less (20%) in phase 2

Table 5 (contd): Full details of studies (n-28) evaluating headache care components associated with quality

Author	Population and setting	Study aim	Methodology	Authors' principal findings/conclusions
Gahir & Larner, 2004 [24]	100 consecutive new headache patients attending a UK neurology service	To examine the use that headache patients had made of the expertise of community pharmacists prior to referral to a neurologist	Cross-sectional study including questions on time since onset of headaches, frequency of GP consultations, number of medications, consultations with a community pharmacist	Most patients (76) had TTH (n=76), 19 migraine and 4 MOH; with headache present for 2 months to >30 years. 52 patients reported 1-5 GP visits (all had ≥1 GP visit as they were referred by GP), 25 reported 6-10 visits, 9 reported 11-20 visits and 14 had made >30 visits. 60 patients had received 3-5 types of medication, and 14 had had >5. 15 had consulted their community pharmacist: for 8, possible treatment or medications were suggested, 3 were advised to see their GP, 1 was warned about side effects. For 4 patients, consultation with pharmacist was not deemed helpful or no advice was given. The community pharmacist had not prescribed medication for any patient.
Gahir & Larner, 2006 [25]	119 patients with headache attending UK outpatient neurology practices	To ascertain proportion of patients with headache seen in general neurology outpatient clinics who have previously been to ED	Cross-sectional study in which patients reported whether they had attended ED. Where possible, patients' reports were corroborated from patients' records.	ED attendance reported by 26 (22%) of patients. 15 were not admitted to hospital and 11 were (9% of all headache patients attending the neurology clinic).

Table 5 (contd): Full details of studies (n-28) evaluating headache care components associated with quality

Author	Population and setting	Study aim	Methodology	Authors' principal findings/conclusions
Harpole *et al*, 2003 [26]	54 headache patients in the US	To evaluate feasibility of a headache management programme and to assess patient outcomes	Prospective study of adult patients referred by GP to a headache management programme. A nurse practitioner delivered educational sessions and diagnosed and treated patients according to guidelines of Headache Guideline Consortium. Patients completed self-administered questionnaires at baseline and 6 months: MIDAS (also at 3 months), SF-36, patient satisfaction instrument and questions on perception of headache management and level of worry about headaches. Additionally, patients' records reviewed for demographics, comorbidities, outpatient and ED visits and inpatient stays in the 6 months before and during study.	Baseline data obtained for all 54 patients; 37 (69%) completed entire survey whilst 12 patients completed only MIDAS at 6 months. Patient satisfaction data available for 26 (48%). 3-month MIDAS completed by 28 (52%). 30% of patients suffered from depression and/or anxiety. Mean MIDAS score was 41, and all SF-36 scores were low. Mean headache frequency was 35 days over 3 months. Patients reported being dissatisfied with their care and very worried about their headaches. MIDAS scores at 6-months had decreased by mean 21.2 ($p<0.05$) and headache frequency by mean 14.5 days over 3 months. 30 (61%) patients had moved to a lower disability grade on MIDAS at 6 months. Improvement on MIDAS at 3 months was similar to that at 6 months. Whilst 67% of patients experienced severe headache-related disability at baseline, only 20% did so at 6 months. Significant improvements were found on 6 of the 8 subscales of SF-36 and the mental health summary score. Patients were significantly more satisfied with their headache care, and reported fewer problems with their headache management and less worry about headaches. The number of ED visits decreased from 0.19 per patients to 0.04 ($p<0.02$). Patients with migraine (51%) were more likely to be treated with triptans than patients without (0%) ($p<0.001$). Only 9% of patients were prescribed prophylactics.<br><br>The authors concluded that the headache management programme is effective as patients experienced significant improvements in headache-related disability and functional status, and patients were more satisfied with their care.

Table 5 (contd): Full details of studies (n-28) evaluating headache care components associated with quality

Author	Population and setting	Study aim	Methodology	Authors' principal findings/conclusions
Harpole *et al*, 2005 [27]	763 headache patients in 3 US health-care practices (community based internal medicine, academic general internal medicine and managed care organization)	To assess headache burden and headache management needs between 3 diverse clinical sites	Survey questionnaire sent to patients with migraine or TTH addressing demographics, headache type, headache-related disability, depression and anxiety, satisfaction with care, general health, worry about headache, problems with headache management and use of health care.	385 of 763 patients (50%) participated. Higher response rate from community (63%) than academic practice (42%) or managed care organization (40%). There were differences in socio-demographic factors between sites but headache characteristics were similar. Mean MIDAS score was 19.1 (range between sites 16.9-23.6, NS). Significant differences between sites for problems with headache management, use of prophylaxis, worry about headache, number of visits to ED, personal expenses for OTC and prescription ad medications. On most of these, scores were higher for managed care site.
Karli *et al*, 2006 [28]	3 GPs evaluated 189 headache patients (89 pre-education and 100 post-education) in Turkey	To investigate impact of 2 days' GP education on diagnostic accuracy and treatment of primary headaches	GPs' diagnosis and treatment of patients compared to headache specialists' ('gold standard'). Specialist diagnoses made by IHS criteria.	GPs' mean diagnostic accuracy improved from 56% pre-education to 81% post education (p<0.001) (range of improvement 11-33%. Agreement rate did not significantly improve for migraine, but for did for TTH (from 40% to 64%; p=0.014). Treatment agreement was analyzed only when GP diagnosis was correct. Pre-education agreement rate 65% (n=31) of patients, post-education 81% (n=65) (p=0.043). Prophylactic treatment prescribed more often post-education (69% *vs* 73%) and acute medication less often (30% *vs* 22%).
Larner, 2005 [29]	114 patients newly referred to a neurology clinic for headache in UK	To ascertain frequency of use of headache guidelines (Migraine in Primary Care Advisors and www.eguidelines.co.uk) in primary care	Patient-reported survey of headache management and consultations prior to referral.	105 patients referred by GP; 22 (21%) reported only one GP consultation prior to referral. Of those who had ≥2 GP consultations, 34 (32%) had been prescribed one or no medication. Total proportion of patients who had seen GP once and been prescribed only one medication was 53%.<br>The authors concluded that, on most optimistic interpretation, only 47% of patients referred to a neurology clinic had been managed according to guidelines.

Table 5 (contd): Full details of studies (n-28) evaluating headache care components associated with quality

Author	Population and setting	Study aim	Methodology	Authors' principal findings/conclusions
Latinovic, Gulliford & Ridsdale, 2006 [30]	413,221 patients aged >15 years who had been diagnosed with headache in 253 general practices in UK over 8 year period	To describe consultation pattern in UK including new and total consultations, and prescription, investigation and referral rates.	Audit of patients whose medical records showed a diagnostic code of 'headache', 'migraine' or 'cephalgia' in the General Practice Research Database (GPRD)	570,793 consultations for headache were made by 413,221 patients. Mean rates were 4.4 consultations/100 registered patients/year (6.39 in women, 2.49 in men). Consultation rates highest between 15 and 24 years of age. 189,065 prescriptions made for antimigraine drugs to 33.9% of patients. Women were more likely to receive prescription than men, and prescribing was most frequent in women aged 45-54 years. Referral data available for 77 practices only: 3,622 referrals had been made to hospital clinical specialties. Rate of referrals was higher in men and highest between 55 and 64 years of age.<br>**Note:** Although the aims state that investigation rates would be described, no results were presented on investigations
Magnusson, Riess & Becker, 2004 [31]	70 patients with transformed migraine and 37 with CDH attending 2 headache clinics in Canada	To compare treatment outcomes in traditional office-based pharmacological treatment programme and multi-disciplinary management programme.	Prospective study at headache clinic and chronic pain clinic. Patients from headache clinic represented pharmacological group (Group 1); patients at chronic pain clinic underwent a multidisciplinary management programme (Group 2). Outcomes assessed by Headache Disability Inventory (HDI), SF-36 and headache diaries.	All patients in Group 1 had diagnosis of transformed migraine and 43 were over-using medication. Only 48 provided baseline and follow up diaries. 17 of these had improvement in headache days per month of ≥25% and 2 worsened by ≥25%, but mean headache severity did not change from baseline (4.6) to follow up (4.5). Mean total HDI score for all 70 patients was 53.4 at baseline and 51.5 at follow up. All subscales of SF-36 were below Canadian norms and had not improved by end of study. In Group 2, 54% had transformed migraine, 9 with MOH, 14% had chronic TTH, 14% headache associated with cervical spine disorders, 8% new daily persistent headache and 8% chronic post-traumatic headache. 36 provided baseline and follow up HDI, 30 provided baseline and follow up SF-36. Mean HDI score was 51.5 at baseline and 34.0 at follow up ($p<0.001$) with 18 of 36 patients showing ≥25% reduction. Mean scores on all 8 sub-scales of SF-36 significantly improved, with greatest improvements in role physical, bodily pain, vitality and social functioning. Mean pain ratings also significantly improved.

Table 5 (contd): Full details of studies (n-28) evaluating headache care components associated with quality

Author	Population and setting	Study aim	Methodology	Authors' principal findings/conclusions
Maizels, 2001 [32]	518 patients who had presented with primary headache at an ED in the US	To evaluate ED care in the 'triptan era'	Medical record audit over 6 months. Only patients who had <3 visits to ED included.	Of 518 patients, 426 (82%) attended ED once and 38 (7%) attended twice. Records could be reviewed for 264 only. Most charts (174) recorded discharge diagnosis of migraine; 90 a non-migraine headache diagnosis. Most patients with non-migraine headache (67) were diagnosed with TTH. An adequate history was recorded for 30 non-migraine patients. Of migraine patients, 11 not previously diagnosed with migraine were diagnosed in ED. Need for prophylactic treatment was identified in40 (31%) patients who were not on it when attending ED. Treatment given was migraine-specific (triptan or ergot) for 46 (26%), non-specific medication justified by contra-indications to triptans for 45 (26%), otherwise non-migraine specific for 43 (25%) and none for 40 (23%).

Michele Peters (University of Oxford, michele.peters@dph.ox.ac.uk), Suraj Perera, Elizabeth Loder, Crispin Jenkinson, Raquel Gil Gouveia, Rigmor Jensen, Zaza Katsarava and Timothy J Steiner

Journal of Headache and Pain

Table 5 (contd): Full details of studies (n-28) evaluating headache care components associated with quality

Author	Population and setting	Study aim	Methodology	Authors' principal findings/conclusions
Maizels, Saenz & Wirjo, 2003 [33]	264 patients with headache identified from ED records or referred by GPs in the US	To evaluate impact of a group-based model of disease management	Prospective open-label observational study of patients attending a group session led by nurse practitioner, with a follow-up consultation. Charts and computer records reviewed to ascertain triptan costs and headache-related visits 6 months pre- and post-intervention. Brief Headache Screen (BHS) used to assess headache frequency at baseline and at 6 months' follow up, used as marker of clinical outcome.	264 patients had attended 25 headache clinics in 1 year and 233 attended a follow-up consultation. Headache data available for 233 patients at baseline and 182 at 6-months' follow up. Most common diagnoses were transformed migraine with medication overuse (n=105) and migraine (n=76). Triptan costs increased by $5,423 (19%) from baseline to follow-up. Headache-related clinic visits reduced from 606 to 413 (31%) and ED visits from 256 to 130 (49%). Nearly all triptan cost increases were accounted for by patients who had not previously been prescribed triptans. Moderate and high triptan users had highest rates of clinic visits pre-intervention, decreasing from 154 to 74 (48%) for moderate users and increasing from 64 to 71 (11%) for high users. Greatest decrease in visits was in the 75 high clinic users at baseline (46%). High clinic users had greatest triptan costs, rising by $20 (16%) from baseline to follow-up. 130 patients did not receive prescriptions for a triptan before or after consultation. These patients had greater reductions in clinic (from 318 to 194; 39%) and ED visits (from 127 to 50; 61%) than patients prescribed triptans. 91 patients reported severe headaches on >2 days per week at baseline. Follow-up data available for 72 (73%), with 62 (82%) of these reporting fewer severe headaches, 55 (76%) reporting severe headaches on ≤2 days per week and 27 (37.5%) having ≤1 severe headache per month. Triptan costs in these patients rose by $949 (9.1%) but headache-related visits decreased from 273 to 189 (31%). 59 (21%) of the patients were recruited through the ED. They accounted for 31% of triptan costs and 46% of headache-related visits at baseline, with triptan costs rising by a mean of $43 (27%), clinic visits decreasing from 276 to 205 (26%) and ED visits decreasing from 185 to 102 (45%). There were no assigned costs in this organization for clinic or ED visits so theoretical costs of $60 and $100 respectively per visit were used. Hence, reduced clinic and ED visits yielded savings of $24,180. Taking into account increased cost of triptans, the programme generated savings of $18,757.

Table 5 (contd): Full details of studies (n-28) evaluating headache care components associated with quality

Author	Population and setting	Study aim	Methodology	Authors' principal findings/conclusions
Matchar *et al*, 2008 [34]	614 patients with primary headaches seen recruited from three different sites: 1) an academic internal medicine practice, 2) a staff-model managed care organization or 3) a community practice in the US	To compare headache disability between patients cared for in a coordinated headache management programme and patients receiving usual care	Randomized controlled trial comparing coordinated management with routine care for headache. The management programme consisted of education session about headache types, triggers and treatment options, diagnosis by a professional trained in headache care and pro-active follow up by a case manager. Patients in control group received usual care from primary care providers. Main outcome measure was MIDAS to compare disability between groups at 6 months. Secondary measures were response at 12 months, general health questions and SF-36, depression (Patient Health Questionnaire Short Form PHQ-9) and satisfaction with headache care.	614 patients randomized into intervention and control groups. 614 surveys received at baseline, 452 at 6 months and 450 at 12 months. 407 patients returned surveys both at 6 and 12 months, 45 at 6 months only, 43 at 12 months only and 119 at neither 6 nor 12 months. MIDAS scores available for 603 patients at baseline, 444 at both 6 and 12 months. 437 patients for whom both baseline and 6 month MIDAS questionnaires were available represented main sample for analysis. At 12 months, complete data were available for 387 patients. Significantly more patients excluded from intervention group (104, 34%) than controls (73, 24%) (p=0.004) because of either failure to return questionnaire or missing data. Excluded patients tended to be younger, with lower mental health scores and more depressive symptoms. The 437 patients in the primary analysis had similar baseline MIDAS scores in intervention (mean 30.8, SD 37.1) and control groups (mean 30.6, SD 42.2). There were no significant differences in age, gender, headache type, insurance type and most SF-36 scales between the groups (significant differences only for role physical, general health and physical component summary score). At 6 months, MIDAS scores were mean 7.7 points lower in intervention than in control group (scores improved by 7-10 in controls and by 12-17 in intervention group (p=0.008). Differential drop out was assessed by last observation carried forward method: the observed intervention effect was not due to differential pattern of drop out. Considering only the intervention group, patients who had attended at least one educational session had a significantly greater improvement in MIDAS score (by mean 17.9) than those who had not attended any classes (3.16). The effect of attending one class was 14.8 points (p=0.016). Secondary outcomes showed SF-36 scores were higher in intervention than in control group, with statistically significant results for role physical, pain, vitality/energy and physical summary score (p<0.05). Better results were also achieved on PHQ-9 and general health questions for intervention group.

Table 5 (contd): Full details of studies (n-28) evaluating headache care components associated with quality

Author	Population and setting	Study aim	Methodology	Authors' principal findings/conclusions
Melchart *et al* 2008 [35]	306 patients with chronic headache admitted to an inpatient unit in one of 3 hospitals in Germany	To describe and compare outcome and quality of care offered by the 3 hospitals, which differed in their treatment concepts: Hospital 1 specialized in treatment of headache with focus on withdrawing painkillers; Hospital 2 had a comprehensive treatment concept including drug and physical therapy as well as complementary therapy; Hospital 3 had a treatment concept based on traditional Chinese medicine.	Prospective observational comparison of outcomes at 4 weeks prior to admission, at admission, discharge and 6 months after discharge. Outcome indicators included pain intensity and frequency, functional ability, depression, quality of life and health-related behavior.	Most patients (92.8%) had migraine; 54.6% had migraine only, 45.4% had TTH with or without migraine. The proportions with MOH were significantly different between hospitals (23% in Hospitals 1 and 2, and 2% in Hospital 3). Significant differences were also found in age, education level, acute medication use, previous experience with complementary therapies and degree of trust in successful treatment, and patients from Hospital 3 had higher average pain intensity, higher headache frequency, higher levels of depression, poorest sense of coherence, lowest health related satisfaction and the poorest food habits. Migraine patients had higher pain intensity, higher disability, lower headache frequency, lower levels of depression and better mental health scores. Patients from all 3 hospitals markedly benefited from treatment at discharge and six months after discharge. At discharge, patients from Hospital 2 rated their treatment more positively than patients from the other hospitals and patients from Hospital 3 were less satisfied with their hospital stay than patients from the other hospitals. Six months after discharge, patients from Hospital 2 showed significantly greater improvements in coping with stress and patients from Hospital 1 were more successful in their use of self-help. Statistically significant differences were found for the migraine sub-group: greater decrease in pain intensity and better overall rating of treatment success at discharge. At six months, patients with TTH (on its own or with migraine) showed higher use of self-help strategies than patients with migraine only. Only slight differences were found in outcomes between the 3 hospitals. There was no significant difference in proportions of patients whose headache frequency was reduced by ≥50%.<br><br>The authors concluded that the results did not suggest a definitive overall ranking between the 3 hospitals.

Table 5 (contd): Full details of studies (n-28) evaluating headache care components associated with quality

Author	Population and setting	Study aim	Methodology	Authors' principal findings/conclusions
Offredy *et al*, 2008 [36]	Patients referred to a redesigned headache service of GPwSI in UK	To pilot a clinical assessment service for headache	Survey of patient satisfaction with clinical assessment service	**Note:** The article predominantly describes development of the clinical assessment service and gives limited information on the satisfaction study. No sample size is given although the figure shows that most patients rated the service as 'good' or 'excellent'.
Ridsdale *et al*, 2008 [37]	61 headache patients receiving care from GPwSI and 56 receiving care in neurology clinic in UK	To compare a newly set up GPwSI headache service to an existing neurology service	A survey to evaluate headache impact, satisfaction and cost.	No significant difference found in headache impact between patients referred to GPwSI and neurology service. However, patients were significantly more satisfied with GPwSI service and costs were lower for GPwSI service.
Soon, Siow & Tan, 2005 [38]	38 migraine patients attending neurologist headache clinic in Singapore	To compare diagnosis, treatment strategies and satisfaction between treatment by community care physicians and at a specialist headache clinic	Observational study, administering questionnaires to patients consulting a neurologist at baseline and 3 months. Questions on diagnosis, treatment strategies, knowledge of migraine treatments and satisfaction with treatment, SF-36 and MIDAS.	Community physician had diagnosed migraine in 32% of patients, whereas neurologist did so in all 38. 63% of patients reported that community physician did not inform them of diagnosis. Neurologist prescribed acute and prophylactic medication more often than community physician (87% *vs* 8%). Neurologist most commonly prescribed triptans as acute therapy and tricyclic antidepressants, anticonvulsants and riboflavin as prophylaxis; community physician mostly prescribed simple analgesics and tricyclic antidepressants. Patients more likely to use alternative therapies after consultations with community physician (35%) than with neurologist (18%). Outcomes significantly improved at 3 months: reductions in headache frequency, pain scores, MIDAS scores and improvements in 5 of the 8 dimensions (physical functioning, role limitations, bodily pain, vitality and social functioning) of SF-36, as well as Mental Health and Physical Health Components of SF-36.

Table 5 (contd): Full details of studies (n-28) evaluating headache care components associated with quality

Author	Population and setting	Study aim	Methodology	Authors' principal findings/conclusions
Vincent & de Carvalho, 1999 [39]	414 headache patients attending a first visit at a specialty clinic in Brazil	To assess accuracy of non-specialist diagnosis, number of investigations and prescriptions of preventative medication	Data from specialists consultations ('gold standard') compared to patient-reported information about non-specialist consultations	Non-specialist diagnosis matching 'gold standard' made for 44.9% of migraine, 6.7% of TTH and 26.7% of cluster headache patients respectively. Patients reported 501 investigations (mean per patient 1.21, range 0-23). Patients had been recommended a mean of 0.6 (SD 1.2) types of prophylactic medications; no preventative treatment had been recommended to 51.1% of migraine, 49.0% of TTH and 33.3% of CH patients.<br><br>The authors concluded that many unnecessary investigations are conducted and that prophylaxis is not used often enough.<br><br>**Note:** They give no information on how many investigations were unnecessary, or on number of patients for whom prophylaxis would have been required.

Table 5 (contd): Full details of studies (n-28) evaluating headache care components associated with quality

Author	Population and setting	Study aim	Methodology	Authors' principal findings/conclusions
Zeeberg, Olesen & Jensen, 2005 [40]	336 patients with and without MOH or probable MOH (pMOH) discharged from a Danish headache centre	To describe clinic procedures and patient characteristics and to evaluate treatment outcomes.	Retrospective audit of patients over 1-year period.	Average treatment period was 7.8 months with mean 5.7 visits. Of 336 patients, 269 did not have MOH or pMOH. Their average headache frequency was 19 days per month for average duration of 16 years. Most common diagnoses were migraine plus TTH (n=73), migraine only (n=60) and chronic TTH only (n=59). Significant reductions in headache frequency (p<0.001) and intensity (p value not stated) achieved for patients with migraine, chronic TTH, frequent episodic TTH and cluster headache. Number of missed workdays due to headache significantly reduced for migraine and frequent episodic TTH (p<0.05). At first visit, 11% used prophylactic medication compared to 39% at discharge.<br><br>67 patients had either MOH (n=35) or pMOH (n=32), with, most commonly, migraine plus TTH (n=33), TTH only (n=20) or migraine only (n=11). pMOH was diagnosed when patients had not fully completed withdrawal or data on headache frequency after withdrawal were missing. Average headache frequency in MOH or pMOH was 27 days per month. At first visit, most widely used medications were simple analgesics (52%), triptans (32%) and combination medication (24%). Significant reductions in headache frequency achieved for migraine (p<0.001) and TTH (p<0.01). At first visit, 13% were on prophylactic medication compared to 51% at discharge. During medication withdrawal, two thirds of patients needed rescue medication whereas one third remained medication free.<br><br>Overall, 32% of patients were referred to a physiotherapist and 10% to a psychologist.

CDH: chronic daily headache; CT: x-ray computerized tomography; ED: emergency department; GP: general practitioner; GPwSI: GP with a special interest; IHS: International Headache Society; MIDAS: Migraine Disability Assessment questionnaire; MOH: medication-overuse headache; MRI: magnetic resonance imaging; NS: not significant; NSAID: non-steroidal anti-inflammatory drug; OTC: over-the counter medication; pMOH: probable medication overuse headache, SD: standard deviation; SF-36: Short-Form-36 Health Survey; TTH: tension-type headache.
